# Loss of strumpellin in the melanocytic lineage impairs the WASH Complex but does not affect coat colour

**DOI:** 10.1111/pcmr.12506

**Published:** 2016-09-12

**Authors:** Benjamin J. Tyrrell, Emma F. Woodham, Heather J. Spence, Douglas Strathdee, Robert H. Insall, Laura M. Machesky

**Affiliations:** ^1^ Cancer Research UK Beatson InstituteCollege of Medical Veterinary and Life SciencesUniversity of GlasgowGlasgowUK

**Keywords:** melanocytes, actin dynamics, migration, vesicle traffic, endocytic trafficking

## Abstract

The five‐subunit WASH complex generates actin networks that participate in endocytic trafficking, migration and invasion in various cell types. Loss of one of the two subunits WASH or strumpellin in mice is lethal, but little is known about their role in mammals in vivo. We explored the role of strumpellin, which has previously been linked to hereditary spastic paraplegia, in the mouse melanocytic lineage. Strumpellin knockout in melanocytes revealed abnormal endocytic vesicle morphology but no impairment of migration in vitro or in vivo and no change in coat colour. Unexpectedly, WASH and filamentous actin could still localize to vesicles in the absence of strumpellin, although the shape and size of vesicles was altered. Blue native PAGE revealed the presence of two distinct WASH complexes, even in strumpellin knockout cells, revealing that the WASH complex can assemble and localize to endocytic compartments in cells in the absence of strumpellin.

**Significance**

The WASH complex is essential for mouse development and plays central roles in endocytic recycling pathways. Here, we show that strumpellin, thought to be a structurally essential component of this complex, is dispensable in murine melanocytes. Our data show that melanocyte migration and melanin production and transfer can occur in the absence of strumpellin. Our results highlight an emerging theme in melanoblast migration that the invasive actin machinery is not as important as the protrusive machinery and call into question the function of strumpellin as an essential core subunit of the WASH complex.

## Introduction

In the adult mouse, mature melanocytes localize to the base of hair follicles where they generate melanin to regulate coat colour. To arrive at the hair follicle niche, melanoblasts, the precursors of melanocytes, undergo a remarkable migration during development. Melanoblasts originate at the neural crest at ~E8.5, and a subset migrate on a dorsal–lateral pathway to populate the skin of the whole embryo by ~E15.5 and ultimately home to hair follicles. Another population of melanoblasts may arise from Schwann cell precursors along peripheral nerves underneath the skin (Adameyko and Lallemend, 2010; Adameyko et al., 2009). Regardless of the origin of melanoblasts, they undergo relatively long‐range persistent cell migration including crossing from the dermis to the epidermis, proliferation and homing to a defined niche. Melanoblasts migrate in the epidermis with long protrusions in a Rac1‐dependent manner, using actin assembly to drive elongation of the cell and translocation between keratinocytes (Li et al., 2011). It is unknown whether melanoblasts also depend on integrin and receptor recycling via the activity of the WASH complex for their migration, as has been reported for other cell types in a 3D environment (Dozynkiewicz et al., 2012; Muller et al., 2009; Zech et al., 2011).

Once melanoblasts mature into melanocytes, they produce melanin and assemble it into granules called melanosomes, which they later transport to surrounding keratinocytes and thus to the nascent hair in mice [reviewed in Yamaguchi and Hearing (2009)]. Melanosomes are lysosome‐related organelles (LROs), which are a specialized vesicular compartment derived by a separate pathway from lysosomes [reviewed in Raposo and Marks (2007)]. Melanosomes and other LROs mature through cargo delivery from early endosomes (Raposo and Marks, 2007). The transport carriers from which melanosomes derive associate with the BLOC‐1 and BLOC‐2 (biogenesis of LRO complexes), which comprise three multiprotein complexes. Mutations in BLOC complex members are implicated in Hermansky–Pudlak syndrome, a genetic disorder manifesting in multiple symptoms including bleeding and albinism. BLOC‐1 interacts with the WASH complex in a network involving the endosomal lipid kinase PI4KIIa (Ryder et al., 2013). BLOC‐1 cargoes such as the Menkes copper transporter ATP7A, which transports copper for tyrosinase enzymatic activities, and the melanosomal SNARE protein VAMP7 (shown to be a BLOC‐1 cargo in neuronal cells) depend on normal WASH complex function, perhaps for their stability, in cultured melanoma cells (Ryder et al., 2013). ATP7A is a BLOC‐1 cargo in melanosomes, where it provides copper transport for tyrosinase activity (Setty et al., 2008). However, the potential roles of the WASH complex are still not clear, as Delevoye et al. (2016) found that recycling endosome tubule formation during melanosome maturation was dependent on ANXA2 (annexin a2) and not WASH.

The WASH complex is a five‐subunit complex, which has been implicated in the generation of actin networks involved in endocytic trafficking of signalling receptors and integrins reviewed in Rotty et al. (2013). WASH is an actin nucleation promoting factor and interacts directly with the Arp2/3 complex to trigger the assembly of branched actin networks. It shows homology to the WASP‐family of proteins, which all have proline‐rich sequences and a c‐terminal VCA domain that binds to Arp2/3. WASH‐generated actin networks are likely to be important for vesicle shaping, including tubulation and/or resolution of tubules (Derivery et al., 2009; Gomez and Billadeau, 2009). The WASH complex associates with retromer and is involved in retrieval of receptors back to the Golgi complex, such as the mannose‐6‐phosphate receptor (Gomez and Billadeau, 2009). WASH has an important function in trafficking of certain receptors, such as the vacuolar ATPase, integrins and EGFR, and the actin networks generated by WASH are also implicated in clustering of receptors and selection of cargo to be rescued from degradation and recycled back to the plasma membrane (Carnell et al., 2011; Duleh and Welch, 2010; Zech et al., 2011, 2012).

The WASH complex subunit strumpellin is a 1599aa protein encoded by the SPG8 (KIAA0196) gene (Valdmanis et al., 2007). Strumpellin is one of over 50 genes mutated in hereditary spastic paraplegias (HSP), which are characterized by corticospinal tract axonopathy [reviewed in Fink (2013)]. The basis for strumpellin's role in HSP is not well understood, as expression of various HSP‐associated mutant strumpellin proteins had no effect on cells in culture (Freeman et al., 2013), suggesting that at least the particular mutations tested did not confer a dominant negative function. Furthermore, mice with only one copy of the strumpellin gene were normal, suggesting that haploinsufficiency was not a likely explanation for HSP phenotypes (Jahic et al., 2015).

The WASH complex is estimated to be 500 kDa, based on an assumed 1:1 stoichiometry of the subunits (strumpellin MW 134 kDa, SWIP (KIAA1033) MW 135 kDa, Fam21MW 146 kDa, WASH MW 51 kDa and CCDC53MW 22 kDa). However, to our knowledge, the size and mobility of the WASH complex have not been measured beyond a previous report that endogenous WASH in cell extracts runs at around 700 kDa on a size exclusion column (Gomez and Billadeau, 2009). Previous studies have also provided evidence for a stable complex where the expression levels of each subunit depend on the other subunits (Derivery et al., 2009; Jia et al., 2010). Strumpellin, Fam21 and SWIP are proposed to be core subunits of the WASH complex, upon which other subunits assemble (Jia et al., 2010). Unlike in mammalian cell cultures, however, in *Dictyostelium*, loss of individual WASH complex subunits did not prevent localization of the others to endocytic vesicles, indicating the potential for at least partial function of subcomplexes or individual subunits (Park et al., 2013).

We have explored the function of strumpellin in the melanocytic lineage both at the cellular and tissue level using tyrosinase‐cre (Tyr::CreB) in a floxed mouse. Tyr::CreB expresses from E11.5, when the melanoblasts have begun to migrate away from the neural tube and are still largely in the dermis (Delmas et al., 2003, 2007). Surprisingly, melanocytes can function normally in the absence of strumpellin even though at the cellular level, there are obvious defects caused by its loss. We also provide evidence that WASH complex subunits can form partial complexes in the absence of strumpellin and that residual WASH proteins localize properly and show actin accumulation at endosomes.

## Results

### Strumpellin knockout in the mouse melanocyte lineage

**Figure 1:**
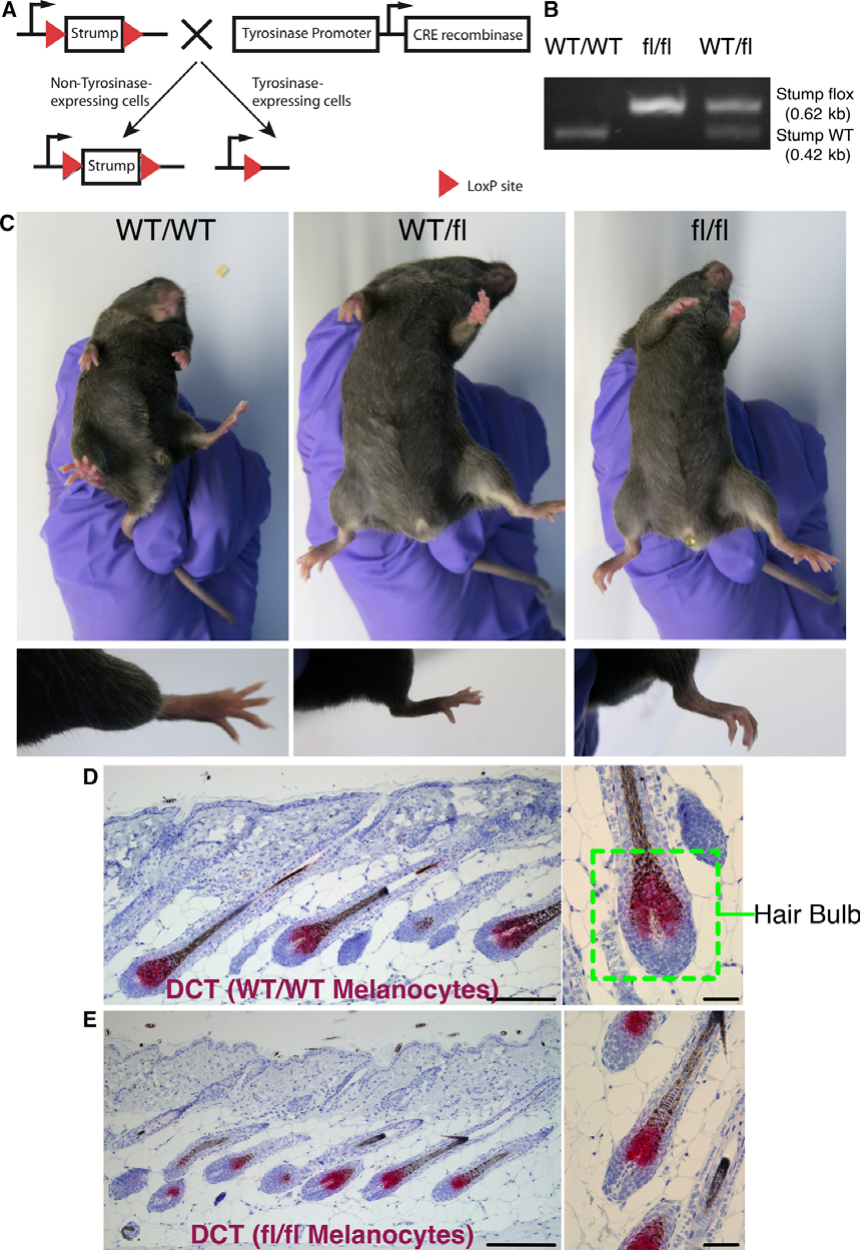
Strumpellin deletion in the melanocyte linage does not impair hair pigmentation or melanocyte localization to hair follicles in adult mice. (A) Gene targeting strategy to generate strumpellin f/f Tyr::CreB^+^ mice. Tyr::CreB excises strumpellin in the melanocyte lineage. (B) A genotyping assay for floxed strumpellin in wild‐type (left lane), strumpellin f/f (middle lane) and strumpellin wt/f (right lane) mice. (C) Coat colour (top) and hindlimb colour (bottom) in ~3‐month‐old wild‐type (left), strumpellin wt/f (middle) and strumpellin f/f (right) Tyr::CreB^+^ mice. (D) Dorsal skin of ~8‐month‐old strumpellin f/f Tyr::CreB^−^ mouse stained by IHC with anti‐DCT (melanocytes) in red at ×10 (left) and ×40 (right) magnification. (E) Dorsal skin of ~8‐month‐old strumpellin f/f Tyr::CreB^+^ mouse stained by IHC with anti‐DCT (melanocytes) in red at ×10 (left) and ×40 (right) magnification. Scale bars for ×10 and ×40 magnification are 200 and 50 μm, respectively.

Previous research on mammalian cells in culture suggested that depletion of Fam21, strumpellin or SWIP leads to destabilization and/or destruction of the entire complex at the protein level (Derivery et al., 2009; Gomez et al., 2012; Jia et al., 2010) similar to the Scar/WAVE complex (Ibarra et al., 2006; Kunda et al., 2003). However, deletion of individual WASH complex subunits in the amoeba *Dictyostelium* resulted in phenotypes unique to the deleted subunit and suggested that remaining subunits could still perform at least partial functions (Park et al., 2013). Here, we ask whether disrupting the WASH complex impairs melanocyte function by knocking out strumpellin specifically in the mouse melanocyte lineage in a C57Bl/6 background. We generated a floxed strumpellin mouse (see Methods) and crossed it onto a Tyr::CreB^+^ (Delmas et al., 2003) in a C57Bl/6 background (Figure 1A). A PCR‐based genotyping assay showed a 0.62 kb PCR product in strumpellin flox/flox (f/f) mice and both 0.62‐ and 0.42‐kb products in strumpellin wild‐type/flox (wt/f) mice (Figure 1B).

Strumpellin knockout in the melanocyte lineage had no apparent effects in young mice; they were healthy, fertile and born at expected Mendelian ratios (Table S1), unlike constitutive strumpellin knockout, which is embryonic lethal (Jahic et al., 2015). Unexpectedly, when strumpellin wt/wt Tyr::CreB^+^ mice were compared with strumpellin f/wt or f/f Tyr::CreB^+^ mice in a C57Bl/6 background, there was no observable difference in coat colour on the abdomen or extremities, including the tail and hind legs (Figure 1C). To test whether there could be consequences for melanocyte stem cells and renewal during the hair cycle, we aged a small cohort of 4 strumpellin wt/wt, 3 wt/f and 6 f/f Tyr::CreB^+^ mice up to 8 months old to determine whether coat colour would grey with time. Again, there was no observable difference in coat colour on abdomen, limbs or tail between the mice (Figure S1A). Additionally, dorsal skin samples showed normal localization of melanocytes to hair follicles in strumpellin wt/wt and f/f Tyr::CreB^+^ mice (Figure 1D, E). The lack of a phenotype was remarkable, not only because constitutive strumpellin knockout is lethal (Jahic et al., 2015), but because of the fundamental role of the WASH complex in invasive migration, vesicle trafficking and cargo sorting (Harbour et al., 2010; Zech et al., 2011, 2012). Additionally, hypopigmentation was observed for BLOC‐1 complex mutant mice (Falcon‐Perez et al., 2002) and BLOC‐1 has been previously biochemically associated with WASH (Ryder et al., 2013). Taken together, our data show that strumpellin is dispensable for differentiation, migration and function of melanoblasts and melanocytes in mice.

### Strumpellin null melanocytes display normal pigment and migration in vitro

**Figure 2:**
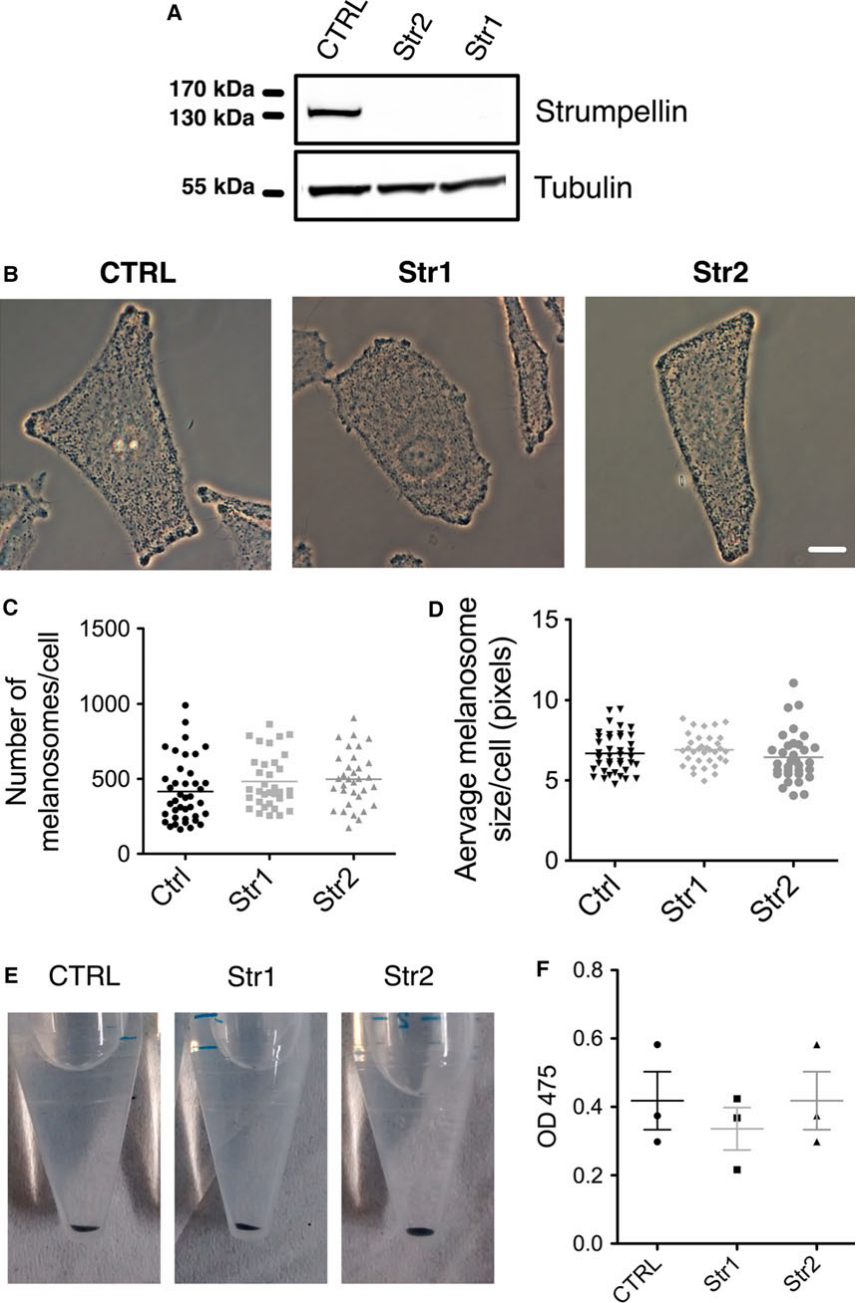
Melanosome number and size are not affected by strumpellin deletion. (A) Western blot showing antistrumpellin and anti‐*α*‐tubulin loading control in strumpellin f/f Tyr::CreB^−^ control (CTRL) or Tyr::CreB^+^ strumpellin knockout (Str1 and Str2) immortal melanocyte line lysates. (B) Phase 3 images of control (CTRL) or Tyr::CreB^+^ strumpellin knockout (Str1 and Str2) immortal melanocyte lines. (C, D) The analysed particle function of ImageJ was used to count the number (C) and size (D) of melanosomes in phase 3 images of control (CTRL) and strumpellin knockout (Str1 and Str2) melanocyte lines (n = 40, 34 and 33 for CTRL, Str1 and Str2, respectively, pooled from three experiments, graphs show means, error bars = SEM, one‐way anova was used for statistical analysis: P = 0.0582 for C and P = 0.1294 for D). (E) Cell pellets of control (CTRL) and strumpellin knockout (Str1 and Str2) immortal melanocyte lines in PBS. (F) OD475 spectrophotometry readings to measure melanin content of 1 × 10^6^ control (CTRL) or strumpellin knockout (Str1 and Str2) immortal melanocyte lines lysed in 1 ml of 1 m NaOH (n = 3, graph shows means, error bars = SEM, one‐way anova was used for statistical analysis: P = 0.7351). Scale bars are 10 μm.

We cultured melanocytes from 1‐day‐old pups to determine whether strumpellin protein was absent in our floxed mouse and whether/how this affected cell and molecular functions. We produced immortalized low‐passage floxed melanocytes by crossing strumpellin f/f Tyr::CreB^+^ mice with Ink4a^−/−^ mice and isolating melanocytes from the skins of 1‐day‐old pups. We isolated three immortal melanocyte lines, a control line from a strumpellin f/f Tyr::CreB^−^ Ink4a^−/−^ mouse (CTRL) and two strumpellin knockout lines from strumpellin f/f Tyr::CreB^+^ Ink4a^−/−^ mice (Str1 and Str2). Western blots of cultured cells confirmed that strumpellin protein was absent from our knockout lines, but present in the control (Figure 2A). We observed no significant difference in melanosome localization, the number of melanosomes per cell or the average melanosome size per cell (Figure 2B–D) regardless of strumpellin expression. Furthermore, the quantity of melanin in terms of cell pellet colour or spectrophotometric measurements of cell lysates was unchanged by strumpellin deletion (Figure 2E, F).

**Figure 3:**
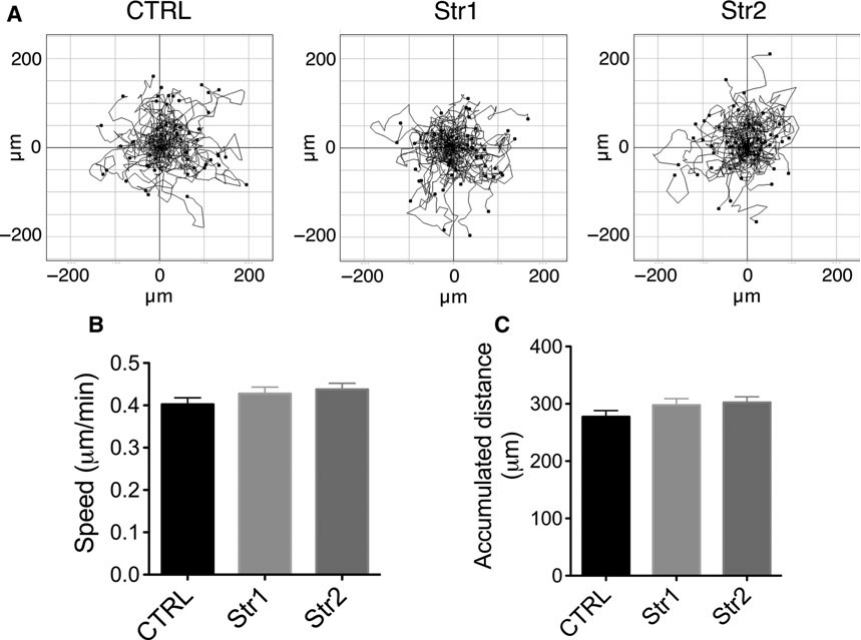
Migration of melanocytes is not dependent on strumpellin. (A) Spider plots for random migration tracks of strumpellin f/f Tyr::CreB^−^ (CTRL) or Tyr::CreB^+^ strumpellin knockout (Str1 and Str2) immortal melanocyte lines. (B and C) The average speed and accumulated distance migrated by strumpellin f/f Tyr::CreB^−^ (CTRL) or Tyr::CreB^+^ strumpellin knockout (Str1 and Str2) immortal melanocyte lines (n = 100 cells, pooled from two experiments, graphs show means, error bars = SEM, one‐way anova was used for statistical analysis: P = 0.2937 and 0.2637 for B and C, respectively).

To assay cell migration, melanocytes were plated at single‐cell density on fibronectin‐coated glass and their movement was tracked over time. Plots of cell trajectories revealed no difference in their migration tracks (Figure 3A), speed or the average accumulated distance, regardless of strumpellin expression (Figure 3B, C). We also saw no difference in the number of focal adhesions in normal versus strumpellin null melanocytes, as marked by vinculin (Figure S2A, B). We conclude that strumpellin was efficiently deleted from melanocytes in our model, but that melanin content and cell migration in vitro were not affected. This largely agrees with our previous studies of WASH knockdown cells and similar studies in *Dictyostelium* WASH knockout amoebas (Carnell et al., 2011).

### Strumpellin knockout melanocytes have enlarged, clustered WASH‐ and WAFL‐positive vesicles

**Figure 4:**
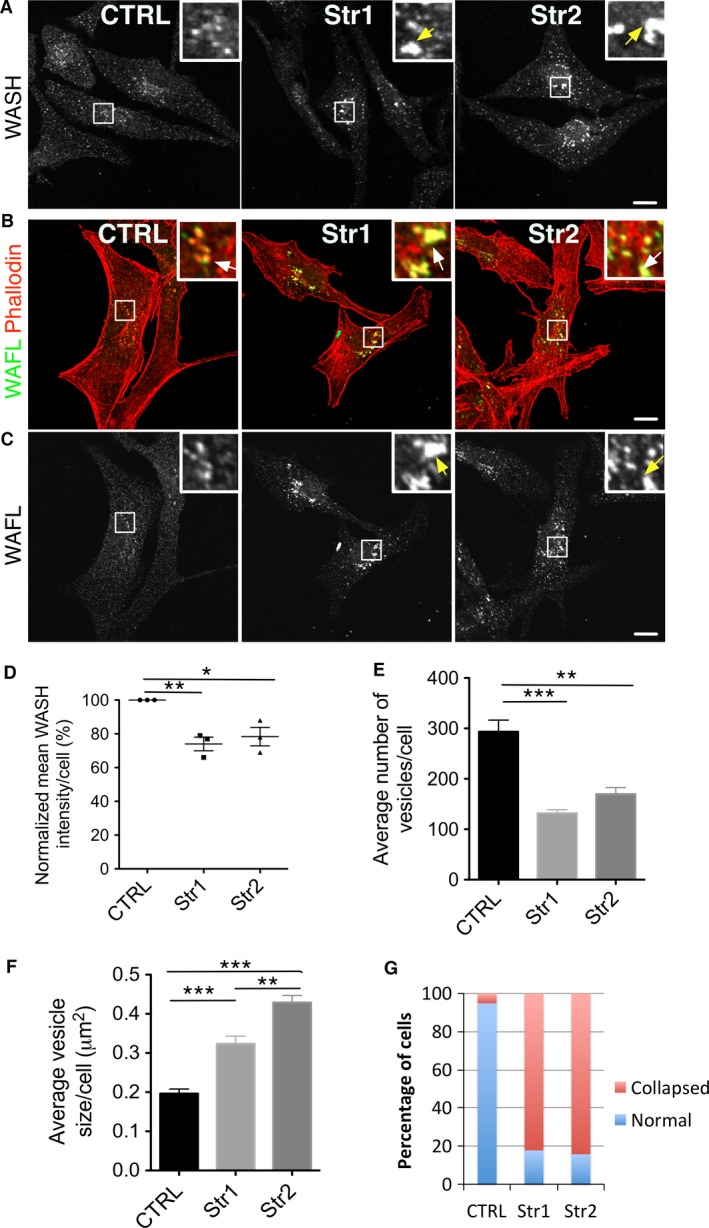
WASH‐ and WAFL‐positive vesicles enlarge and cluster near the cell centre in strumpellin knockout melanocytes. (A) Strumpellin f/f Tyr::CreB^−^ (left – CTRL) or Tyr::CreB^+^ strumpellin knockout (middle and right – Str1 and Str2) immortal melanocyte lines were immunostained against anti‐WASH, and inserts are enlargements of the perinuclear region and show enlarged WASH‐positive vesicles (arrows). (B) Strumpellin f/f Tyr::CreB^−^ control (left – CTRL) or Tyr::CreB^+^ strumpellin knockout (middle and right – Str1 and Str2) immortal melanocyte lines were immunostained against anti‐WAFL (green) and phalloidin (red), and inserts are enlargements of the perinuclear region and show colocalization between WAFL and actin (arrows). (C) Single‐channel WAFL image from B. Arrows show collapsed and enlarged WAFL‐positive vesicles present in strumpellin f/f Tyr::CreB^+^ immortal melanocyte lines. (D) The measure function of ImageJ was used to quantify the fluorescent intensity of whole Control (CTRL) or strumpellin knockout (Str1 and Str2) immortal melanocyte line cells immunostained against anti‐WASH (n = 3, a total of 59, 44 and 57 CTRL, Str1 and Str2 cells, respectively, with at least 10 cells per experimental repeat). Graph show means, error bars = SEM, one‐way anova was used for statistical analysis: P = <0.0072, Dunn's multiple comparison post‐test compared all columns for statistical difference: *P ≤ 0.05, **P = ≤0.01) (E). Control (CTRL) and strumpellin knockout (Str1 and Str2) immortal melanocyte lines stained with anti‐WAFL were scored blind for clustered and enlarged WAFL‐positive vesicles (n = 97, 62 and 51 for CTRL, Str1 and Str2 cells, respectively, pooled from three experiments). (F, G). The analysed particle function of ImageJ was used to count the number of WASH‐positive vesicles (F) and measure WASH‐positive vesicle size (G) in control (CTRL) and strumpellin knockout (Str1 and Str2) immortal melanocyte lines (n = 64, 62 and 42 for CTRL, Str1 and Str2, respectively, pooled from three experiments, graphs show means, error bars = SEM, one‐way anova was used for statistical analysis: P = <0.0001 for both F and G, Dunn's multiple comparison post‐test compared all columns for statistical difference: *P ≤ 0.05, **P = ≤0.01, ***P ≤ 0.001). Scale bars are 10 μm.

We next queried whether loss of strumpellin from melanocytes affected WASH‐positive vesicles. Immunostaining of subconfluent immortal melanocytes cultured from 1‐day‐old pups (as described above) revealed that WASH and the associated protein WAFL [also known as FKBP15 (Harbour et al., 2012; Pan et al., 2010)] were expressed and, more importantly, localized to vesicles in strumpellin knockout cells (Figure 4A–C), despite the validated absence of strumpellin protein. Interestingly, some of the WASH‐ and WAFL‐positive puncta of strumpellin knockout melanocytes appeared enlarged and clustered in the perinuclear region (Figure 4A–C, insert arrows). While WASH was still localized to puncta resembling endocytic vesicles in strumpellin null cells, we measured an overall ~30% decrease in WASH fluorescence intensity in strumpellin nulls (Figure 4D). We determined the average number and size of WASH‐positive vesicles in cells using ImageJ (Schindelin et al., 2012). The analysis revealed a marked decrease in average vesicle number per cell from 293 in control (CTRL) cells to 131 and 169 in Str1 and Str2 cells, respectively (CTRL versus Str1 P = ≤0.001, CTRL versus Str2 P = ≤0.01, Str1 versus Str2 P = >0.05) (Figure 4E), and an increase in average vesicle size from 0.20 μm^2^ in CTRL to 0.32 and 0.43 μm^2^ in Str1 and Str2 cells, respectively (CTRL versus Str1 P = ≤0.001, CTRL versus Str2 P = ≤0.001, Str1 versus Str2 P = ≤0.01) (Figure 4F). We saw a similar change in WAFL distribution, as over 80% of cells from both strumpellin knockout melanocyte lines displayed enlarged, clustered perinuclear WAFL‐positive vesicles compared to less than 10% in the control melanocytes (Figure 4G). In summary, strumpellin‐depleted melanocytes displayed enlarged perinuclear vesicles containing WASH and WAFL, two components of WASH‐positive vesicles in normal cells. Strumpellin thus does not appear to be important for the localization of WASH, filamentous actin or WAFL to these vesicles, but it is important for their normal morphology and subcellular localization.

**Figure 5:**
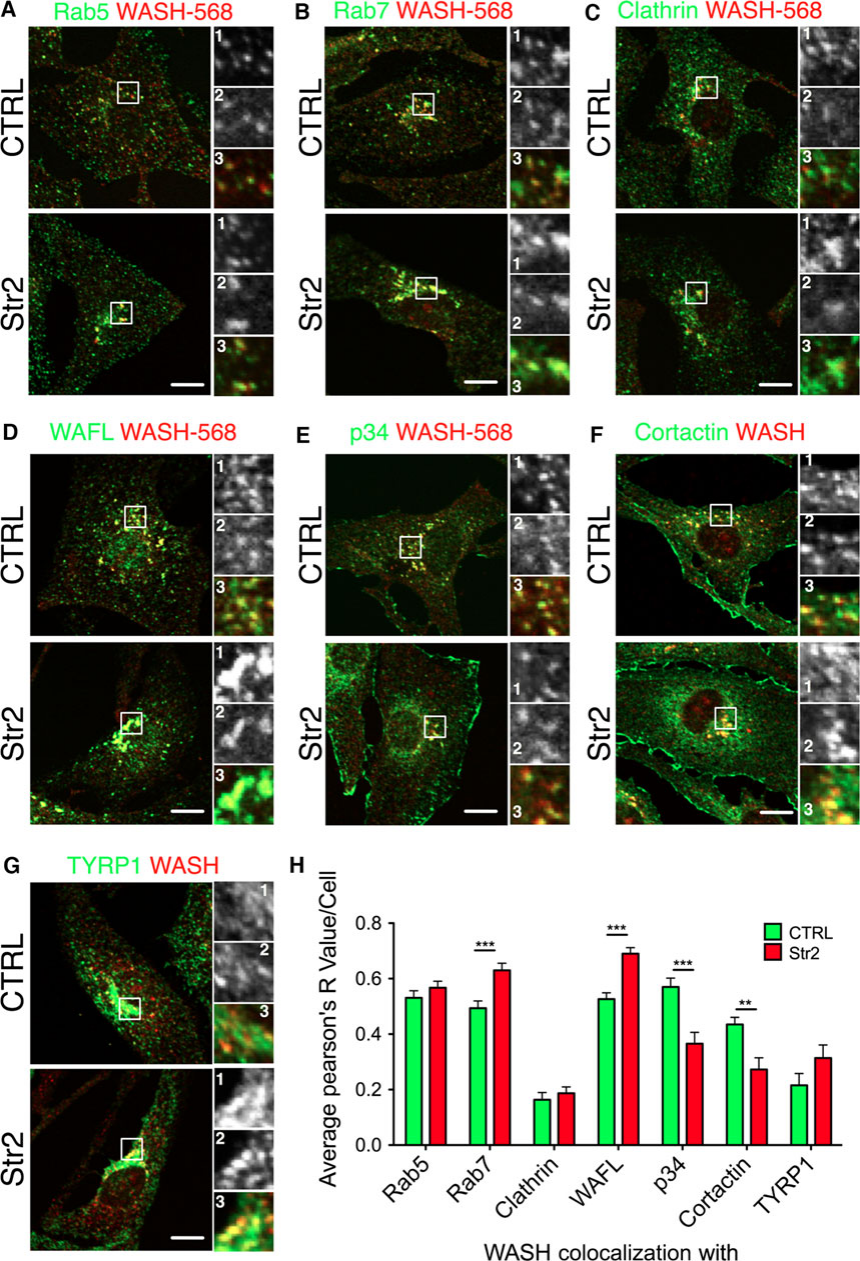
Wash colocalization with endocytic and cytoskeletal markers is altered in strumpellin knockout melanocytes. (A–E) Control (CTRL) and strumpellin knock out (Str2) immortal melanocyte lines were immunostained with anti‐WASH‐alexa568‐conjugated antibody and costained with Rab5 (A), Rab7 (B), clathrin (C), WAFL (D) or p34 (Arp2/3 complex) (E). Enlarged perinuclear regions show the green channel (labelled ‘1’), the red channel (labelled ‘2’) and the merged image (labelled ‘3’). (F, G) Control (CTRL) and strumpellin knock out (Str2) immortal melanocyte lines were immunostained with anti‐WASH antibody and cortactin (F) or TYRP1 (G). Enlarged perinuclear regions show the green channel (labelled ‘1’), the red channel (labelled ‘2’) and the merged image (labelled ‘3’). (H) The Pearson's R value was used to measure colocalization between WASH and various markers (see Methods) in control (CTRL) and strumpellin knockout (Str2) immortal melanocyte lines (n = 30 cells for each condition pooled from three experiments, graphs show means, error bars = SEM,* t* test was used for statistical analysis: P = 0.296 for Rab5, P = 0.0004 for Rab7, P = 0.4994 for clathrin, P < 0.0001 for WAFL, P = 0.0002 for p34, P = 0.0054 for cortactin, P = 0.3027 for TYRP1 colocalization with WASH. **P ≤ 0.01, ***P ≤ 0.001). Scale bars are 10 μm.

As WASH localizes to both early and late endosomes (Derivery et al., 2009; Duleh and Welch, 2010, 2012; Zech et al., 2011), we queried the identity of the WASH‐positive compartment(s) in strumpellin knockout cells. To investigate whether strumpellin loss affects WASH localization to defined vesicle compartments, we costained melanocytes with WASH and Rab5 (early endosome), Rab7 (late endosome), clathrin, WAFL, p34 (Arp2/3 complex, branched actin), cortactin (branched actin) or TYRP1 (melanosome) (Figure 5A–G) and measured colocalization using the Pearson's R value (Figure 5H). Localization to Rab5‐positive early endosomes was unchanged in strumpellin knockout cells (Figure 5A, H); however, there was a significant increase in the colocalization of WASH and the late endosomal marker Rab7 (Figure 5B, H). There was minimal colocalization between WASH and the endocytosis marker clathrin, which was unchanged in strumpellin knockout melanocytes (Figure 5C, H). Furthermore, WASH and WAFL showed strong colocalization that increased significantly in strumpellin knockout melanocytes (Figure 5D, H). As we still observed actin on WASH‐positive vesicles in the strumpellin null cells, we asked whether the associated actin nucleation machinery was also present, in the form of p34 (Arp2/3 complex) and cortactin, a branched actin‐associated protein. Interestingly, there was a decrease in the colocalization between both p34 and cortactin with WASH in strumpellin null cells (Figure 5E, F, H); however, measurable colocalization was still apparent. Finally, given the association of WASH with the melanosome biogenesis BLOC‐1 complex (Ryder et al., 2013), we also asked whether melanosome organization was altered in strumpellin knockout cells. Staining against TYRP1, which marks melanosomes, and WASH showed no obvious change in melanosome organization between strumpellin expressing and knockout melanocytes; WASH‐positive vesicles in strumpellin knockout melanocytes consistently showed a low level of association with melanosomes (Figure 5G, H).

Thus, we conclude that although the morphology of the WASH‐positive compartment is altered, both the early and late endocytic compartments still colocalize with WASH in strumpellin null cells. Additionally, the actin nucleation machinery is still present on these compartments, but at a reduced level. WASH association with melanosomes is minimal and appears largely unaltered in strumpellin knockout melanocytes.

### Strumpellin is not essential for association of other WASH complex subunits

**Figure 6:**
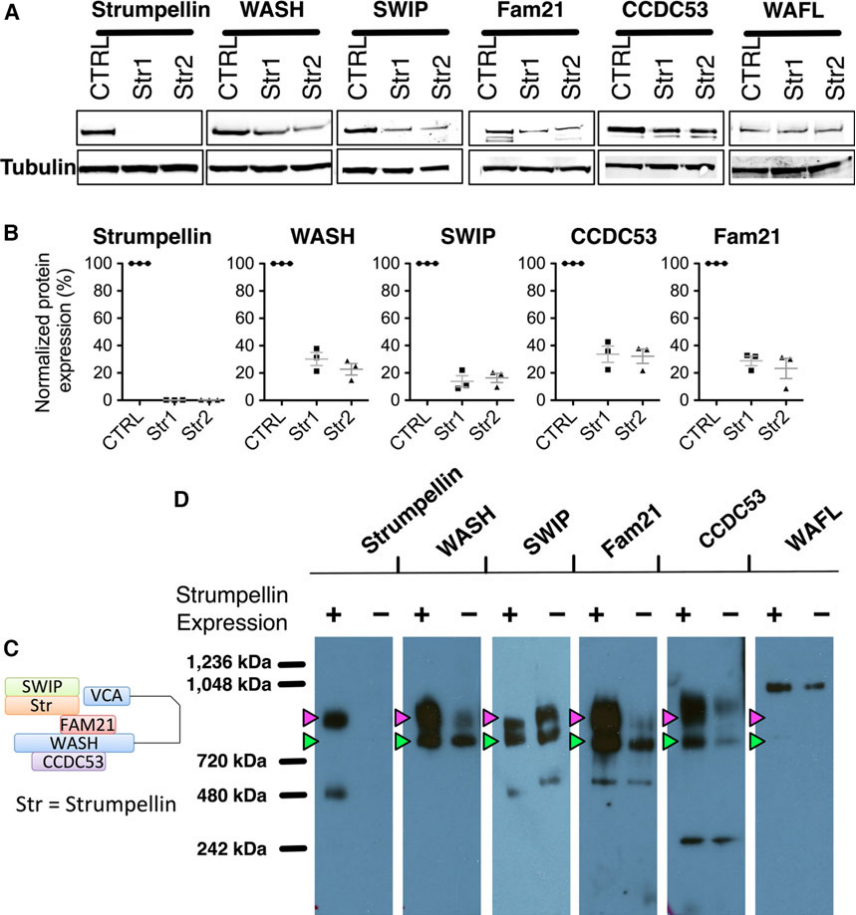
Melanocytes exhibit two high molecular weight complexes containing WASH, only the higher one of which comigrates with strumpellin. (A) Western blot against antistrumpellin, anti‐WASH, anti‐SWIP, anti‐Fam21, anti‐CCDC53 and anti‐WAFL with anti‐*α*‐tubulin loading control of control (CTRL) or strumpellin knockout (Str1 and Str2) immortal melanocyte line lysates. (B) Integrated density analysis of strumpellin, WASH, SWIP, CCDC53 and Fam21 expression in strumpellin knockout immortal melanocyte line lysates (Str1 and Str2) normalized against control (CTRL) immortal melanocyte line lysates (n = 3). (C) Schematic representation of the WASH complex (based on Jia et al., 2010). SWIP is green, Strumpellin (Str) is orange, Fam21 is red, WASH is shown with its VCA domain folded back into the complex in blue and a connecting line between the domains, and CCDC53 is purple. Protein sizes are not to scale. (D) Blue native PAGE Western blot showing antistrumpellin, anti‐WASH, anti‐SWIP, anti‐Fam21, anti‐CCDC53 and anti‐WAFL in control (CTRL) or strumpellin knockout (Str1) immortal melanocyte line lysates.

While loss of strumpellin did not disrupt WASH localization to early or late endocytic compartments, it was unclear whether other WASH complex subunits remained intact and associated. We performed Western blots to probe for strumpellin, WASH, SWIP, Fam21, CCDC53 and WAFL in cell lysates of confluent control and strumpellin knockout immortal melanocytes. Strumpellin expression was completely abolished in knockout cells, as shown by the lack of a band at ~130 kDa (Figure 6A, B). WASH migrates at ~60 kDa on Western blots and was still expressed in strumpellin knockout cells but at a reduced level of ~30%, SWIP migrates at ~130 kDa at a reduced level of ~20%, CCDC53 migrates at ~35 kDa at a reduced level of ~30% and Fam21 migrates above 170 kDa at a reduced level of ~30% (Figure 6A, B). Finally, WAFL migrates at ~170 kDa on Western blots and WAFL expression was unchanged following strumpellin knockout (Figure 6A). Full Western blots for Figure 6(A) are shown in Figure S2(C).

WASH forms a putative 500‐kDa core complex, together with four other subunits (Fam21, strumpellin and WASH‐interacting protein SWIP, strumpellin and coiled‐coil domain‐containing protein 53 CCDC53) (Figure 6C) (Jia et al., 2010). WASH‐containing complexes were purified by conventional chromatography from HeLa cells, but the stoichiometry of the subunits and the size of the complex was not addressed (Jia et al., 2010). To address whether the WASH expressed in strumpellin knockout melanocytes was part of a complex, we performed blue native PAGE, a method to separate native protein complexes on acrylamide gels based on size (Wittig et al., 2006). We compared the mobilities of components of the WASH complex and the associated WAFL protein in immortal melanocytes with and without strumpellin (CTRL and Str1 lines) (Figure 6D). We successfully probed our native PAGE blots for strumpellin, WASH, SWIP, Fam21, CCDC53 and WAFL. In control cells, all members of the WASH complex comigrated as two large complexes, Figure 6(D) magenta and green arrowheads. These complexes were between the 720‐ and 1048‐kDa markers except for strumpellin, which only comigrated with the top complex (magenta arrowhead). A few of the subunits displayed some lower minor bands possibly representing smaller or partial complexes: strumpellin at 480 kDa, SWIP and Fam21 between 480 and 720 kDa and CCDC53 at ~242 kDa. WAFL migrated as a single band near ~1048 kDa that was larger than any of the WASH complex members (Figure 6D). In the absence of strumpellin, the other four WASH complex members still comigrated as two high molecular weight complexes and WAFL migration was unchanged. The smaller of the two complexes was more abundant for WASH and Fam21 in strumpellin knockout cells, and there was an even distribution of SWIP and CCDC53 between the two complexes in the strumpellin knockout cells (Figure 6D). Thus, the WASH complex in melanocytes may have two forms, one of which lacks strumpellin, or alternatively, strumpellin may be more loosely associated with the WASH complex and thus partially dissociated during the native PAGE preparation. An association between WASH and the other subunits still occurs in the absence of strumpellin, even though the levels of most of the subunits are diminished in strumpellin knockout cells. Furthermore, WAFL migrates at a larger size than either of the two WASH complexes, indicating that WAFL is not part of a stable complex with WASH in melanocytes. We conclude that strumpellin is not required for co‐association of the other WASH complex subunits (WASH, SWIP, Fam21 and CCDC53) and that WASH complex subunits may form two distinct stable complexes in melanocytes.

## Discussion

The remarkable journey that melanoblasts undertake during development and their subsequent differentiation into melanin‐producing melanocytes [reviewed in Mort et al. (2015)] remains an area of active research (Mort et al., 2016). The WASH complex has been speculated to be a potential regulator of pigmentation, but here, we find that strumpellin is dispensable for melanoblast and melanocyte migration in vivo and in vitro as well as for pigmentation of the mouse hair. To the best of our knowledge, there are no homologues to strumpellin in the mouse genome, so it is unlikely that another protein compensated for strumpellin loss.

Strumpellin has been linked to hereditary spastic paraplegia (Fink, 2013) and to the BLOC‐1 complex (Ryder et al., 2013), so it is remarkable that mice lacking strumpellin in the melanocytic lineage displayed normal coat colour. However, most of the mutations in strumpellin that have been associated with HSP are missense changes and so would likely result in partial protein function (Valdmanis et al., 2007). (Ryder et al. (2013) showed that depletion of strumpellin from MNT‐1 melanoma cells caused a missorting of BLOC‐1 cargoes ATP7A and VAMP7 to the plasma membrane. However, our in vivo studies suggest that strumpellin is not limiting for WASH complex function in melanocytes, or perhaps that missorting defects of strumpellin null cells were mild enough not to prevent normal hair pigmentation. These data agree with the recent finding that ANXA2 and not WASH was important for recycling endosomal tubule formation during melanosome maturation (Delevoye et al., 2016). It could be interesting to knock out WASH from the melanocyte lineage and determine whether residual WASH complex function was responsible for the mild phenotype of the strumpellin nulls or whether WASH was not required at all for pigmentation.

The skin of adult C57Bl/6 mice lacking strumpellin in melanocytes showed normal localization of melanocytes to hair follicles, and mice had normal coat colour up to at least 1 yr of age. The normal black pigmentation of the coat implies that strumpellin is not limiting for the melanoblast journey from the neural tube to the skin and hair follicles, or for the formation and survival of melanocyte stem cells in the hair follicles. Our previous work highlighted a role for the small GTPase Rac1 and the actin bundling protein fascin‐1 in epidermal melanoblast migration in vivo (Li et al., 2011; Ma et al., 2013). Furthermore, the constitutive knockout of the Ras‐ and Rac‐associated adapter protein lamellipodin also impaired melanoblast migration, as did the knock‐down of the SCAR/WAVE complex in zebrafish (Law et al., 2013). Finally, deletion in melanocytes of the actin nucleation promoting protein N‐WASP, an Arp2/3 activator in invasive protrusions, did not affect melanoblast migration in vivo (Li et al., 2011). We conclude that migration of melanoblasts does not require the N‐WASP‐ and WASH‐dependent invasive actin machinery for efficient migration in vivo, but this migration is more dependent on dynamic actin protrusions, including long pseudopodia containing filopodia and lamellipodia. Alternatively, depletion of strumpellin leaves enough residual WASH complex activity to support the integrin and receptor functions of WASH. Resolution of this will require further investigation and functional studies.

Strikingly, at the cellular level, we saw an abnormal morphology of the early and late endocytic compartments in strumpellin knockout melanocytes, demonstrating that loss of strumpellin did have functional consequences in melanocytes at least in vitro. While the WASH complex subunits (WASH, SWIP, Fam21, CCDC53 – Figure 6) were only expressed at around 30% of normal levels, WASH appeared to localize normally to early endosomes with an increased colocalization to late endosomes, as seen for *α*5*β*1 integrin in WASH knockdown cells (Zech et al., 2011). A similar abnormal endocytic vesicle morphology was observed in WASH knockout MEFs (Gomez et al., 2012), but a key difference was the presence of filamentous actin clusters on the abnormal vesicles in strumpellin knockout melanocytes, but not WASH knockout MEFs (Gomez et al., 2012). Furthermore, we observed both the Arp2/3 complex and cortactin present on WASH‐positive vesicles in these cells, although colocalization with WASH was reduced when compared to control cells. Thus, the partial WASH complex in strumpellin knockout melanocytes can still likely assemble branched actin networks. Interestingly, as noted in the WASH knockout MEFs (Gomez et al., 2012), we saw no evidence of the increased vesicle tubulation that was apparent in WASH knock‐down experiments (Derivery et al., 2009; Harbour et al., 2010). It is unclear why the endocytic abnormality in strumpellin knockout melanocytes occurs, as the remaining WASH complex still localizes to endosomes and appears capable of inducing actin nucleation on these endosomes. This might imply that partial WASH complexes lacking strumpellin or low levels of WASH complex overall are defective in export tubules/vesicles, leading to an accumulation in the early and late endocytic compartments.

Both WASH and the WASH complex‐associated protein WAFL localized to abnormal endocytic vesicles in strumpellin knockout melanocytes. Our data agree with strumpellin knockout data from *Dictyostelium* that showed localization of GFP‐tagged WASH, SWIP, CCDC53 and FAM21 to vesicles in the absence of strumpellin (Park et al., 2013). Through the use of blue native PAGE, we have shown that not only are WASH and the other members of the WASH complex expressed in the absence of strumpellin but co‐associate in a multiprotein complex. Intriguingly, the wild‐type WASH complex migrates as two distinct bands, both larger than 500 kDa. This could be due to altered stoichiometry of one or more subunits, altered mobility on native PAGE or the presence of components that have not been previously described. To our surprise, strumpellin is only detected in the larger complex. SWIP was found in both complexes and did not change much in the absence of strumpellin, while CCDC53 has significantly diminished its comigration with both subcomplexes in the knockout cells. Additional methods would be needed to study these protein complexes to explain these observations.

WAFL migrated at a larger size than either of the WASH complexes. WAFL has been shown to associate with both WASH complexes (Freeman et al., 2014; Harbour et al., 2010, 2012; Pan et al., 2010) and with retromer (Harbour et al., 2010, 2012; Zavodszky et al., 2014), and it has been argued that retromer mediates the recruitment of both WASH and WAFL to endosomes (Harbour et al., 2010). WAFL may thus comigrate with retromer and while both the WASH complex and WAFL colocalize to endosomes and interact with each other, they are not in a stable complex. It remains to be determined what the upper and lower WASH complexes represent.

In conclusion, knockout of strumpellin in the melanocyte lineage revealed that strumpellin is dispensable for melanoblast migration in vivo and for adult mouse coat colour. Strumpellin knockout melanocytes show abnormal endosome morphology, but WASH still localizes and colocalizes with filamentous actin on endocytic vesicles. Our study suggests that strumpellin is at least partly dispensable for WASH complex function and may be readily detachable from the other subunits of the WASH complex in vivo.

## Methods

### Generation of a conditional allele of the strumpellin gene in mice

All methods using mice in this study were carried out in accordance with UK Home Office Regulations. An ES cell clone (HEPD0534‐7‐C01) for the conditional knockout of the strumpellin gene (E430025E21Rik^tm1a(EUCOMM)Hmgu^) was obtained from the IKMC repository (Skarnes et al., 2011). Cells were cultured using standard protocols on DR4‐irradiated MEF monolayers (Tucker et al., 1997).

Appropriate targeting of the strumpellin gene in the ES cell clone was confirmed by screening the 5′ (GCTAGTGTCAGCGACAGAGTGCGCGCTC and CACAACGGGTTCTTCTGTTAGTCC) and 3′ sides (TCTATAGTCGCAGTAGGCGG and CAGGTTCACTGGCAGGTCGTGCTGTTAGAC) by PCR using Expand Long Template PCR (Roche, West Sussex, UK) with the oligos indicated according to manufacturer's instructions. The presence of the isolated loxP site was also confirmed by PCR (TCATTCCCAGCACCTGTGTC and TGAACTGATGGCGAGCTCAG). Following confirmation of correct targeting, mouse lines were derived by injection of strumpellin ES cells into C57BL/6J blastocysts according to standard protocols (Nagy et al., 2003). Resultant chimeric mice were identified by their agouti coat colour.

After breeding of chimeras, germline‐transmitting chimeras were identified by the coat colour of their offspring and the presence of the modified allele was confirmed by genotyping with the 3′ loxP primers described above. Heterozygous mice were subsequently crossed with a mouse line expressing Flpe (Tg(ACTFLPe)9205Dym) to delete the selectable marker by recombination at the FRT sites (Rodriguez et al., 2000). Correct removal of the lacZ gene and selectable marker cassette was validated by PCR across the remaining FRT site (CCAGAGGTGTGGCTCATAGG and TGACTGTTGGACAGGACACG). Following removal of the cassette exon, the modified allele has 2 loxP sites remaining flanking exon 12 (Ensembl ID: ENSMUSE00000222520 in mouse genome assembly GRCm38.p4) of the strumpellin gene. Following Cre recombination, exon 12 is deleted resulting in premature termination of mRNA translation.

### Generation of immortal melanocyte cell lines

Immortal melanocyte cell lines were created as in (Li et al., 2011), briefly 1‐day‐old strumpellin f/f Ink4a‐Arf^−/−^ mice that were positive (Str1 and Str2) or negative (CTRL) for Tyr::CreB were sacrificed and a skin sample was cut into small pieces using scissors and forceps, incubated in 2 ml of collagenase type 1 and 4 (Sigma, Dorset, UK), centrifuged and resuspended in cell dissociation buffer (Gibco, Thermo Fisher, Loughborough, UK), repeatedly passed through 18*g* and then 20*g* needles, centrifuged, left to settle in wash buffer, centrifuged again and resuspended in melanocyte growth media (F‐12 media (Invitrogen, Thermo Fisher, Loughborough, UK) containing 10% FBS, 200 nm TPA (Sigma) and 100 μg/ml primocin (InvivoGen, Tolouse, France). Medium was changed every 3 days, and cells were spilt at 50–70% confluence. After 3 days, Geneticin (Gibco) was added to the medium at 50 μg/ml for 3 days of each week, and once a week, cells were washed in PE (PBS 1 mm EDTA) for 10 min with occasional gentle agitation. Over 3–4 weeks, this resulted in a pure immortal melanocyte cell line. Experiments were performed on cells between 2 and 4 months old.

### Immunohistochemistry

At ~8 months old, mice were sacrificed using a UK Home Office Schedule 1 asphyxiation method, dorsal skin was shaved and dissected onto filter paper, cut into strips, fixed in neutral‐buffered 10% formalin overnight and mounted in paraffin blocks. Sections from the blocks were deparaffinized and rehydrated by passage through xylene and a decreasing concentration alcohol series. Antigen retrieval was achieved by the use of microwave‐heated citrate buffer (Dako, Ely, UK) in a pressure cooker. Sections were blocked in 10% rabbit serum for 1 h and incubated for 2 h in goat anti‐DCT (D‐18) antibody (Santa Cruz, Heidelberg, Germany). Sections were washed in PBS then incubated in rabbit anti‐goat biotinylated secondary antibody for 1 h and washed in PBS. The signal was increased using Vectastain ABC kit (Vector Laboratories, Inc.) and visualized with alkaline phosphatase substrate kit (Vector Laboratories, Inc., Peterborough, UK). Counterstaining with haematoxylin revealed cell nuclei.

### Determination of melanin content by spectrophotometry

Immortal melanocytes cultured to 80% confluence were trypsinized and counted. About 1 × 10^6^ cells were sedimented in a 15‐ml falcon tube, resuspended in PBS and sedimented again. The pellet was resuspended in 1 ml of 1 m NaOH at room temperature for 15 min. Relative melanin concentration was determined by OD475 measurements of the resulting suspension using a Beckman Coulter DU 720 spectrophotometer.

### Random migration assays

Fibronectin was diluted in PBS to 10 μg/ml and used to coat glass bottom 6‐well plates for 2 h at 37°C. Coated dishes were washed in PBS. Immortal melanocytes were trypsinized and seeded at 50 000 cells per well of a 6‐well plate and left to adhere overnight. Images of cells were taken every 30 min for 24 h using a Nikon TiE timelapse microscope. Individual cells were tracked using the mTrackJ plugin for ImageJ, and spider plots were generated using the Chemotaxis Tool plugin for ImageJ.

### Immunofluorescence

For immunofluorescence, cells were washed in PBS and fixed for 20 min in 4% formaldehyde in PBS. Cells were permeabilized in 1% NP40 alternative (Calbiochem, Millipore, West Lothian, UK) in PBS for 3 min and blocked in 10% goat serum in PBS for 30 min and then incubated with various combinations of primary antibodies, 1/400 rabbit anti‐WASH4P (Atlas Antibodies – HPA002689, Stockholm, Sweden), 1/400 rabbit anti‐FKBP135 (WAFL) antibody (Abcam – ab14432, Cambridge, UK), 1/100 rabbit anti‐Rab5 (C8B1) (CST – 3547), 1/50 rabbit anti‐Rab7 (D95F2) XP (CST – 9367), 1/100 rabbit anti‐clathrin heavy chain (D3C6) XP (CST – 4796), 1/200 rabbit anti‐p34arc/ARPC2 (Millipore 07‐227, West Lothian, UK), 1/200 mouse anticortactin (p80/85) clone 4F11 (Millipore 05‐180), 1/200 mouse antivinculin (Sigma V9131) and/or mouse anti‐TYRP1 [TA99] antibody (Abcam ab3312) for 1 h at room temperature washed with PBS then incubated for 30 min in 1/1000 goat anti‐rabbit Alexa 488 or 1/1000 goat anti‐rabbit Alexa 568 (Molecular Probes) and/or 1/1000 goat anti‐mouse Alexa 488 or 1/200 phalloidin‐Alexa 568 (Life Technologies, Thermo Fisher, Loughborough, UK). Samples were washed in PBS and mounted onto microscope slides using ProLong Gold antifade reagent with DAPI (Molecular Probes, Thermo Fisher, Loughborough, UK). Images were taken at fixed exposure levels on an Olympus FV1000 or Nikon A1R confocal microscope.

To enable the costaining of rabbit anti‐WASH with other rabbit antibodies for colocalization analysis (Figure 5), an anti‐WASH‐568 conjugate was generated (Molecular probes Alexa Fluor^®^ 568 Antibody Labeling Kit – Alexa 568 was conjugated directly to the antibody as per the manufacturer's instructions) and staining was performed sequentially. First samples were labelled with rabbit antibody ‘X’ as above and then washed extensively in PBS followed by incubation with 1/6 rabbit anti‐WASH‐568 conjugate for 1 h at room temperature and washed in PBS before mounting as above.

### Vesicle analysis

To quantify the number and size of WASH‐positive vesicles, we used the ‘analysed particle’ function of ImageJ. Images of cells stained against WASH were generated, and individual cells were picked out using the ImageJ selection tool and processed as follows. The ImageJ filter subtract background was applied with a rolling ball radius of 20 pixels. A threshold of the image that showed individual vesicles was generated. This threshold setting was determined on an individual experiment basis and applied to all images in that experiment. The ImageJ binary ‘watershed’ filter was applied to the image. Finally, the analysed particle function was applied to count the number and size of WASH‐positive vesicles, using a size limitation of 0.05‐infinity pixels and circularity of 0–1. Outlines of this analysis were generated and overlaid to the original image to ensure accurate vesicle identification. The same procedure was used to quantify the number and size of melanosomes in immortal melanocytes that had been imaged using a ×100 phase3 objective on a Zeiss Axio Observer A1, except a subtract background step was not required and analysed particle parameters were set to a size limitation of 1‐infinity pixels and circularity of 0–1.

### WASH intensity analysis and colocalization analysis

For WASH intensity analysis, fixed exposure images of cells immunostained with anti‐WASH were outlined using the ROI tool in ImageJ and the fluorescent intensity for the entire cell was calculated using the ‘measure’ function in ImageJ. For colocalization analysis, the subtract background function of imageJ was applied to each channel of the image individually and using only the WASH channel image a 10 × 10 μm box was placed over the strongest signal at the cell centre. The Coloc 2 plugin for ImageJ was run using this ROI, and the resulting Pearson ‘R’ value was used as a measure of WASH colocalization with various markers.

### SDS‐PAGE

Melanocytes in a dish were lysed in RIPA buffer (150 mm NaCl, 10 mm Tris pH 7.5, 1 mm EDTA, 1% Triton X‐100, 0.1% SDS) with Halt protease inhibitor (Pearce, Thermo Fisher, Loughborough, UK) for 10 min on ice. Samples were spun at 20 000*g* for 10 min at 4°C, and pellets were discarded. Equal protein, measured using Precision Red (Cytoskeleton, Cambridge, UK), was separated by SDS‐PAGE and transferred to nitrocellulose membranes. Membranes were blocked in 5% BSA PBS‐T for 1 h at room temperature, incubated on a rocker overnight at 4°C with 1/1000 rabbit anti‐WASH (Millipore ABS72), 1/500 rabbit anti‐SWIP (Proteintech 51101‐1‐AP, Manchester, UK), 1/500 rabbit anti‐Fam21C (Millipore ABT79), 1/500 rabbit anti‐CCDC53 (Millipore ABT69), 1/2000 rabbit antistrumpellin (Abcam ab101222) or 1/2000 rabbit anti‐FKBP135 (WAFL) (Abcam ab14432) and 1/20 000 mouse anti‐alpha‐tubulin (Sigma T6199) loading control in 5% BSA PBS‐T. Blots were washed in PBS‐T and then incubated with 1/10 000 goat anti‐mouse Alexa 680 and 1/10 000 goat anti‐rabbit Dylight^™^ 800 4× PEG (Life Technologies) in 5% BSA PBS‐T. Blots were imaged on a Li‐Cor ODYSSEY^®^ CLx. Protein band integrated density analysis was performed using image studio lite software (LI‐COR, https://www.licor.com/bio/products/software/image_studio_lite/) and normalized to 100% in control cells.

### Blue native PAGE

NativePAGE^™^ Sample Prep Kit (Thermo Scientific – BN2008, Thermo Fisher, Loughborough, UK) was used to prepare BN‐PAGE samples. Each dish of melanocytes was lysed in 400 μl of NP‐lysis buffer [100 μl of NP‐buffer, 260 μl of ddH_2_O, 40 μl 5% digitonin, Halt protease inhibitor (Pearce)] for 10 min on ice and scraped with a cell scraper. Samples were centrifuged at 20 000*g* for 30 min at 4°C, and pellets were discarded. Samples of equal protein, determined using Precision Red (Cytoskeleton), were separated by BN‐PAGE NativePAGE^™^ Novex^™^ 3–12% Bis‐Tris Protein Gels from Thermo Scientific as per the manufacturer's instructions (‘NativePAGE^™^ Bis‐Tris Gel protocol’ from Thermo Scientific) with one lane containing NativeMark^™^ Unstained Protein Standard (ThermoFisher Scientific) as a molecular weight standard. Protein was transferred from the gels onto PVDF membranes and fixed for 15 min in 8% acetic acid. Membranes were blocked in 5% milk for 1 h at room temperature and incubated overnight rocking at 4°C in 5% milk PBS‐T with 1/500 rabbit anti‐WASH (Millipore ABS72), rabbit anti‐SWIP (Proteintech 51101‐1‐AP), rabbit anti‐Fam21C (Millipore ABT79), rabbit anti‐CCDC53 (Millipore ABT69), rabbit antistrumpellin (Abcam ab101222) or rabbit anti‐FKBP135 (WAFL) (Abcam ab14432). Membranes were washed in PBS‐T and incubated for 1 h with 1/1000 goat anti‐rabbit horseradish peroxidase‐conjugated secondary antibody (Cell Signalling, Hitchin, UK). Super Signal West Pico Chemiluminescent substrate (Thermo Scientific) was used to generate a signal that was detected using Fujifilm SuperRX X‐ray Film (Bedfordshire, UK). The film was developed on a Kodak X‐OMAT 3000RA (Carestream, Hemel Hempstead, UK) processor and scanned to generate a digital copy using an Epson Perfection 4870 Photo scanner (Epson UK Ltd., Hemel Hempstead, UK).

## Supporting information


**Figure S1.** Strumpellin knockout in the melanocyte linage does not impair coat colour in adult mice.

**Figure S2.** Focal adhesion number is unaffected by strumpellin knockout in melanocytes.

**Table S1.** The genotype and number of mice born from (strumpellin WT/f Tyr::CreB^+^) crossed with (strumpellin WT/f Tyr::CreB^+^).

## References

[pcmr12506-bib-0001] Adameyko, I. , and Lallemend, F. (2010). Glial versus melanocyte cell fate choice: Schwann cell precursors as a cellular origin of melanocytes. Cell. Mol. Life Sci. 67, 3037–3055. 20454996 10.1007/s00018-010-0390-y PMC11115498

[pcmr12506-bib-0002] Adameyko, I. , Lallemend, F. , Aquino, J.B. et al. (2009). Schwann cell precursors from nerve innervation are a cellular origin of melanocytes in skin. Cell 139, 366–379. 19837037 10.1016/j.cell.2009.07.049

[pcmr12506-bib-0003] Carnell, M. , Zech, T. , Calaminus, S.D. , Ura, S. , Hagedorn, M. , Johnston, S.A. , May, R.C. , Soldati, T. , Machesky, L.M. , and Insall, R.H. (2011). Actin polymerization driven by WASH causes V‐ATPase retrieval and vesicle neutralization before exocytosis. J. Cell Biol. 193, 831–839. 21606208 10.1083/jcb.201009119 PMC3105540

[pcmr12506-bib-0004] Delevoye, C. , Heiligenstein, X. , Ripoll, L. et al. (2016). BLOC‐1 brings together the actin and microtubule cytoskeletons to generate recycling endosomes. Curr. Biol. 26, 1–13. 26725201 10.1016/j.cub.2015.11.020 PMC4713302

[pcmr12506-bib-0005] Delmas, V. , Martinozzi, S. , Bourgeois, Y. , Holzenberger, M. , and Larue, L. (2003). Cre‐mediated recombination in the skin melanocyte lineage. Genesis 36, 73–80. 12820167 10.1002/gene.10197

[pcmr12506-bib-0006] Delmas, V. , Beermann, F. , Martinozzi, S. et al. (2007). Beta‐catenin induces immortalization of melanocytes by suppressing p16INK4a expression and cooperates with N‐Ras in melanoma development. Genes Dev. 21, 2923–2935. 18006687 10.1101/gad.450107 PMC2049194

[pcmr12506-bib-0007] Derivery, E. , Sousa, C. , Gautier, J.J. , Lombard, B. , Loew, D. , and Gautreau, A. (2009). The Arp2/3 activator WASH controls the fission of endosomes through a large multiprotein complex. Dev. Cell 17, 712–723. 19922875 10.1016/j.devcel.2009.09.010

[pcmr12506-bib-0008] Dozynkiewicz, M.A. , Jamieson, N.B. , Macpherson, I. et al. (2012). Rab25 and CLIC3 collaborate to promote integrin recycling from late endosomes/lysosomes and drive cancer progression. Dev. Cell 22, 131–145. 22197222 10.1016/j.devcel.2011.11.008 PMC3507630

[pcmr12506-bib-0009] Duleh, S.N. , and Welch, M.D. (2010). WASH and the Arp2/3 complex regulate endosome shape and trafficking. Cytoskeleton (Hoboken) 67, 193–206. 20175130 10.1002/cm.20437 PMC2887680

[pcmr12506-bib-0010] Duleh, S.N. , and Welch, M.D. (2012). Regulation of integrin trafficking, cell adhesion, and cell migration by WASH and the Arp2/3 complex. Cytoskeleton (Hoboken) 69, 1047–1058. 23012235 10.1002/cm.21069 PMC3582321

[pcmr12506-bib-0011] Falcon‐Perez, J.M. , Starcevic, M. , Gautam, R. , and Dell'angelica, E.C. (2002). BLOC‐1, a novel complex containing the pallidin and muted proteins involved in the biogenesis of melanosomes and platelet‐dense granules. J. Biol. Chem. 277, 28191–28199. 12019270 10.1074/jbc.M204011200

[pcmr12506-bib-0012] Fink, J.K. (2013). Hereditary spastic paraplegia: clinico‐pathologic features and emerging molecular mechanisms. Acta Neuropathol. 126, 307–328. 23897027 10.1007/s00401-013-1115-8 PMC4045499

[pcmr12506-bib-0013] Freeman, C. , Seaman, M.N. , and Reid, E. (2013). The hereditary spastic paraplegia protein strumpellin: characterisation in neurons and of the effect of disease mutations on WASH complex assembly and function. Biochim. Biophys. Acta 1832, 160–173. 23085491 10.1016/j.bbadis.2012.10.011 PMC3714738

[pcmr12506-bib-0014] Freeman, C.L. , Hesketh, G. , and Seaman, M.N. (2014). RME‐8 coordinates the activity of the WASH complex with the function of the retromer SNX dimer to control endosomal tubulation. J. Cell Sci. 127, 2053–2070. 24643499 10.1242/jcs.144659 PMC4004978

[pcmr12506-bib-0015] Gomez, T.S. , and Billadeau, D.D. (2009). A FAM21‐containing WASH complex regulates retromer‐dependent sorting. Dev. Cell 17, 699–711. 19922874 10.1016/j.devcel.2009.09.009 PMC2803077

[pcmr12506-bib-0016] Gomez, T.S. , Gorman, J.A. , De Narvajas, A.A. , Koenig, A.O. , and Billadeau, D.D. (2012). Trafficking defects in WASH‐knockout fibroblasts originate from collapsed endosomal and lysosomal networks. Mol. Biol. Cell 23, 3215–3228. 22718907 10.1091/mbc.E12-02-0101 PMC3418315

[pcmr12506-bib-0017] Harbour, M.E. , Breusegem, S.Y. , Antrobus, R. , Freeman, C. , Reid, E. , and Seaman, M.N. (2010). The cargo‐selective retromer complex is a recruiting hub for protein complexes that regulate endosomal tubule dynamics. J. Cell Sci. 123, 3703–3717. 20923837 10.1242/jcs.071472 PMC2964111

[pcmr12506-bib-0018] Harbour, M.E. , Breusegem, S.Y. , and Seaman, M.N. (2012). Recruitment of the endosomal WASH complex is mediated by the extended ‘tail’ of Fam21 binding to the retromer protein Vps35. Biochem. J. 442, 209–220. 22070227 10.1042/BJ20111761

[pcmr12506-bib-0019] Ibarra, N. , Blagg, S.L. , Vazquez, F. , and Insall, R.H. (2006). Nap1 regulates *Dictyostelium* cell motility and adhesion through SCAR‐dependent and ‐independent pathways. Curr. Biol. 16, 717–722. 16581519 10.1016/j.cub.2006.02.068

[pcmr12506-bib-0020] Jahic, A. , Khundadze, M. , Jaenisch, N. et al. (2015). The spectrum of KIAA0196 variants, and characterization of a murine knockout: implications for the mutational mechanism in hereditary spastic paraplegia type SPG8. Orphanet J. Rare Dis. 10, 147. 26572744 10.1186/s13023-015-0359-x PMC4647479

[pcmr12506-bib-0021] Jia, D. , Gomez, T.S. , Metlagel, Z. , Umetani, J. , Otwinowski, Z. , Rosen, M.K. , and Billadeau, D.D. (2010). WASH and WAVE actin regulators of the Wiskott–Aldrich syndrome protein (WASP) family are controlled by analogous structurally related complexes. Proc. Natl Acad. Sci. USA 107, 10442–10447. 20498093 10.1073/pnas.0913293107 PMC2890800

[pcmr12506-bib-0022] Kunda, P. , Craig, G. , Dominguez, V. , and Baum, B. (2003). Abi, Sra1, and Kette control the stability and localization of SCAR/WAVE to regulate the formation of actin‐based protrusions. Curr. Biol. 13, 1867–1875. 14588242 10.1016/j.cub.2003.10.005

[pcmr12506-bib-0023] Law, A.L. , Vehlow, A. , Kotini, M. et al. (2013). Lamellipodin and the Scar/WAVE complex cooperate to promote cell migration in vivo. J. Cell Biol. 203, 673–689. 24247431 10.1083/jcb.201304051 PMC3840943

[pcmr12506-bib-0024] Li, A. , Ma, Y. , Yu, X. et al. (2011). Rac1 drives melanoblast organization during mouse development by orchestrating pseudopod‐driven motility and cell‐cycle progression. Dev. Cell 21, 722–734. 21924960 10.1016/j.devcel.2011.07.008 PMC3464460

[pcmr12506-bib-0025] Ma, Y. , Li, A. , Faller, W.J. , Libertini, S. , Fiorito, F. , Gillespie, D.A. , Sansom, O.J. , Yamashiro, S. , and Machesky, L.M. (2013). Fascin 1 is transiently expressed in mouse melanoblasts during development and promotes migration and proliferation. Development 140, 2203–2211. 23633513 10.1242/dev.089789 PMC3912869

[pcmr12506-bib-0026] Mort, R.L. , Jackson, I.J. , and Patton, E.E. (2015). The melanocyte lineage in development and disease. Development 142, 1387. 25804742 10.1242/dev.123729 PMC4378253

[pcmr12506-bib-0027] Mort, R.L. , Ross, R.J. , Hainey, K.J. , Harrison, O.J. , Keighren, M.A. , Landini, G. , Baker, R.E. , Painter, K.J. , Jackson, I.J. , and Yates, C.A. (2016). Reconciling diverse mammalian pigmentation patterns with a fundamental mathematical model. Nat. Commun. 7, 10288. 26732977 10.1038/ncomms10288 PMC4729835

[pcmr12506-bib-0028] Muller, P.A. , Caswell, P.T. , Doyle, B. et al. (2009). Mutant p53 drives invasion by promoting integrin recycling. Cell 139, 1327–1341. 20064378 10.1016/j.cell.2009.11.026

[pcmr12506-bib-0029] Nagy, A. , Gertsenstein, M. , Vintersten, K. , and Behringer, R. (2003). Manipulating the Mouse Embryo: A Laboratory Manual. (Cold Spring Harbor, NY: Cold Spring Harbor Laboratory).

[pcmr12506-bib-0030] Pan, Y.F. , Viklund, I.M. , Tsai, H.H. , Pettersson, S. , and Maruyama, I.N. (2010). The ulcerative colitis marker protein WAFL interacts with accessory proteins in endocytosis. Int. J. Biol. Sci. 6, 163–171. 20376207 10.7150/ijbs.6.163 PMC2850539

[pcmr12506-bib-0031] Park, L. , Thomason, P.A. , Zech, T. , King, J.S. , Veltman, D.M. , Carnell, M. , Ura, S. , Machesky, L.M. , and Insall, R.H. (2013). Cyclical action of the WASH complex: FAM21 and capping protein drive WASH recycling, not initial recruitment. Dev. Cell 24, 169–181. 23369714 10.1016/j.devcel.2012.12.014

[pcmr12506-bib-0032] Raposo, G. , and Marks, M.S. (2007). Melanosomes–dark organelles enlighten endosomal membrane transport. Nat. Rev. Mol. Cell Biol. 8, 786–797. 17878918 10.1038/nrm2258 PMC2786984

[pcmr12506-bib-0033] Rodriguez, C.I. , Buchholz, F. , Galloway, J. , Sequerra, R. , Kasper, J. , Ayala, R. , Stewart, A.F. , and Dymecki, S.M. (2000). High‐efficiency deleter mice show that FLPe is an alternative to Cre‐loxP. Nat. Genet. 25, 139–140. 10835623 10.1038/75973

[pcmr12506-bib-0034] Rotty, J.D. , Wu, C. , and Bear, J.E. (2013). New insights into the regulation and cellular functions of the ARP2/3 complex. Nat. Rev. Mol. Cell Biol. 14, 7–12. 23212475 10.1038/nrm3492

[pcmr12506-bib-0035] Ryder, P.V. , Vistein, R. , Gokhale, A. , Seaman, M.N. , Puthenveedu, M.A. , and Faundez, V. (2013). The WASH complex, an endosomal Arp2/3 activator, interacts with the Hermansky–Pudlak syndrome complex BLOC‐1 and its cargo phosphatidylinositol‐4‐kinase type IIalpha. Mol. Biol. Cell 24, 2269–2284. 23676666 10.1091/mbc.E13-02-0088 PMC3708732

[pcmr12506-bib-0036] Schindelin, J. , Arganda‐Carreras, I. , Frise, E. et al. (2012). Fiji: an open‐source platform for biological‐image analysis. Nat. Methods 9, 676–682. 22743772 10.1038/nmeth.2019 PMC3855844

[pcmr12506-bib-0037] Setty, S.R. , Tenza, D. , Sviderskaya, E.V. , Bennett, D.C. , Raposo, G. , and Marks, M.S. (2008). Cell‐specific ATP7A transport sustains copper‐dependent tyrosinase activity in melanosomes. Nature 454, 1142–1146. 18650808 10.1038/nature07163 PMC2812007

[pcmr12506-bib-0038] Skarnes, W.C. , Rosen, B. , West, A.P. et al. (2011). A conditional knockout resource for the genome‐wide study of mouse gene function. Nature 474, 337–342. 21677750 10.1038/nature10163 PMC3572410

[pcmr12506-bib-0039] Tucker, K.L. , Wang, Y. , Dausman, J. , and Jaenisch, R. (1997). A transgenic mouse strain expressing four drug‐selectable marker genes. Nucleic Acids Res. 25, 3745–3746. 9278500 10.1093/nar/25.18.3745 PMC146932

[pcmr12506-bib-0040] Valdmanis, P.N. , Meijer, I.A. , Reynolds, A. et al. (2007). Mutations in the KIAA0196 gene at the SPG8 locus cause hereditary spastic paraplegia. Am. J. Hum. Genet. 80, 152–161. 17160902 10.1086/510782 PMC1785307

[pcmr12506-bib-0041] Wittig, I. , Braun, H.P. , and Schagger, H. (2006). Blue native PAGE. Nat. Protoc. 1, 418–428. 17406264 10.1038/nprot.2006.62

[pcmr12506-bib-0042] Yamaguchi, Y. , and Hearing, V.J. (2009). Physiological factors that regulate skin pigmentation. BioFactors 35, 193–199. 19449448 10.1002/biof.29 PMC2793097

[pcmr12506-bib-0043] Zavodszky, E. , Seaman, M.N. , Moreau, K. , Jimenez‐Sanchez, M. , Breusegem, S.Y. , Harbour, M.E. , and Rubinsztein, D.C. (2014). Mutation in VPS35 associated with Parkinson's disease impairs WASH complex association and inhibits autophagy. Nat. Commun. 5, 3828. 24819384 10.1038/ncomms4828 PMC4024763

[pcmr12506-bib-0044] Zech, T. , Calaminus, S.D. , Caswell, P. , Spence, H.J. , Carnell, M. , Insall, R.H. , Norman, J. , and Machesky, L.M. (2011). The Arp2/3 activator WASH regulates alpha5beta1‐integrin‐mediated invasive migration. J. Cell Sci. 124, 3753–3759. 22114305 10.1242/jcs.080986 PMC3225265

[pcmr12506-bib-0045] Zech, T. , Calaminus, S.D. , and Machesky, L.M. (2012). Actin on trafficking: Could actin guide directed receptor transport? Cell Adh. Migr. 6, 476–481. 23076144 10.4161/cam.21373 PMC3547890

