# Supplementary figures and images for: Loss of strumpellin in the melanocytic lineage impairs the WASH Complex but does not affect coat colour
Source: Pigment Cell Melanoma Res. 2016 Sep 12;29(5):559–71. doi: 10.1111/pcmr.12506 (PMC5082549; doi:10.1111/pcmr.12506)

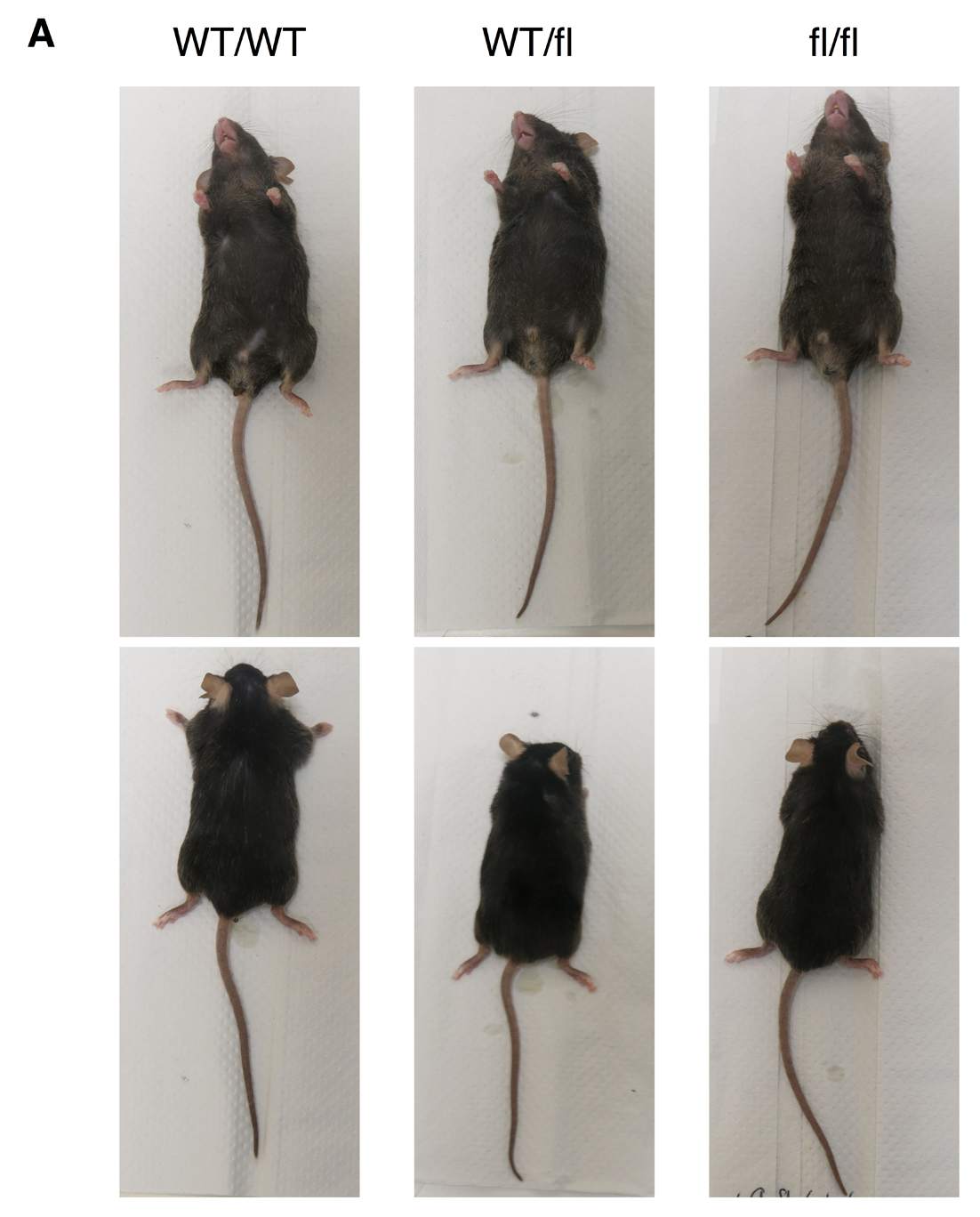

Supplement: Supplementary file 1 — Figure S1. Strumpellin knockout in the melanocyte linage does not impair coat colour in adult mice. [file PCMR-29-559-s001.tif]

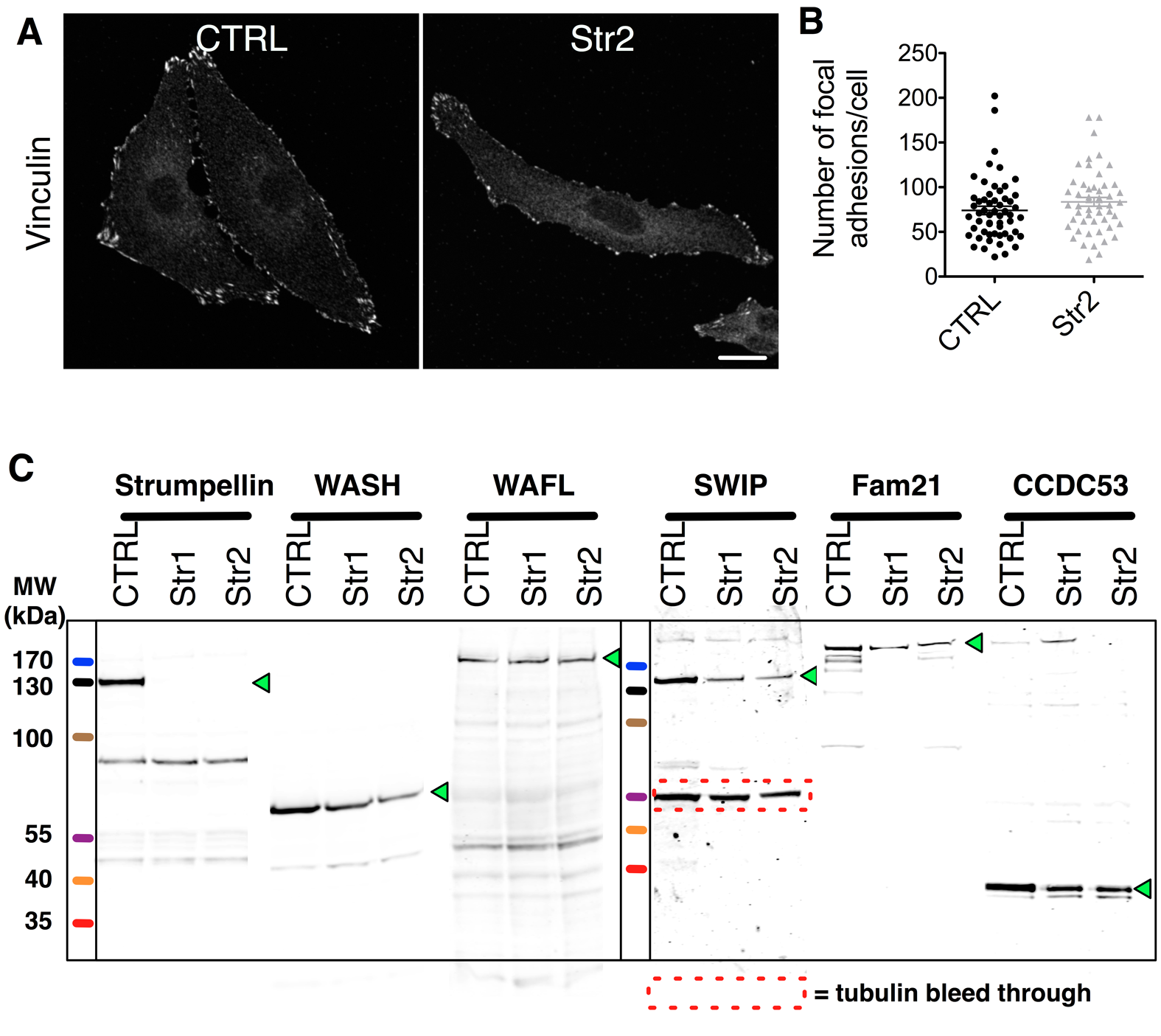

Supplement: Supplementary file 2 — Figure S2. Focal adhesion number is unaffected by strumpellin knockout in melanocytes. [file PCMR-29-559-s002.tif]
