# Supplementary material for: Loss of strumpellin in the melanocytic lineage impairs the WASH Complex but does not affect coat colour
Source: Pigment Cell Melanoma Res. 2016 Sep 12;29(5):559–71. doi: 10.1111/pcmr.12506 (PMC5082549; doi:10.1111/pcmr.12506)
Supplement: Supplementary file 3 — Table S1. The genotype and number of mice born from (strumpellin WT/f Tyr::CreB+) crossed with (strumpellin WT/f Tyr::CreB+). [file PCMR-29-559-s003.docx]

**Tyrrell et al. Supplementary Information**

**Supplementary Figure S1. Strumpellin knockout in the melanocyte linage does not impair coat colour in adult mice.** (A) Dorsal and ventral Coat colour (top and bottom respectively) of ~8 month old wildtype (left), strumpellin wt/f (middle) and strumpellin f/f (right) Tyr::CreB^+^ mice.

**Supplementary Figure S2.** **Focal adhesion number is unaffected by strumpellin knockout in melanocytes.** Strumpellin f/f Tyr::CreB^-^ control (CTRL) or Tyr::CreB^+^ strumpellin knockout (Str2) immortal melanocyte lines were immunostained against anti-vinculin. (B) The number of focal adhesions per cell in control (CTRL) or strumpellin knockout (Str2) immortal melanocyte lines were counted (N=56 and 52 cells pooled from 3 experiments for CTRL and Str2 repectively, graphs show means, error bars = SEM, t-test was used for statistical analysis: P=0.1364). (C) Full western blots for strumpellin, WASH, WAFL, SWIP, Fam21 and CCDC53, arrowed bands are shown in figure 6A. Scale bar is 10μm.

Table S1: The genotype and number of mice born from (strumpellin WT/f Tyr::CreB+) crossed with (strumpellin WT/f Tyr::CreB+).

|  | wt/wt | wt/flox | flox/flox |
| --- | --- | --- | --- |
| Number of mice | 18 | 44 | 14 |
